# North Atlantic warming during Dansgaard-Oeschger events synchronous with Antarctic warming and out-of-phase with Greenland climate

**DOI:** 10.1038/srep20535

**Published:** 2016-02-05

**Authors:** Tine L. Rasmussen, Erik Thomsen, Matthias Moros

**Affiliations:** 1 CAGE- Centre for Arctic Gas Hydrate, Environment and Climate, Department of Geology, UiT Arctic University of Norway, N-9037 Tromsø, Norway; 2 Department of Geoscience, University of Aarhus, DK-8000 Aarhus C, Denmark; 3 Leibniz Institute for Baltic Sea Research Warnemünde (IOW), D-18119 Rostock-Warnemünde, Germany

## Abstract

The precise reason for the differences and out-of-phase relationship between the abrupt Dansgaard-Oeschger warmings in the Nordic seas and Greenland ice cores and the gradual warmings in the south-central Atlantic and Antarctic ice cores is poorly understood. Termed the bipolar seesaw, the differences are apparently linked to perturbations in the ocean circulation pattern. Here we show that surface and intermediate-depth water south of Iceland warmed gradually synchronously with the Antarctic warming and out of phase with the abrupt warming of the Nordic seas and over Greenland. The hinge line between areas showing abrupt and gradual warming was close to the Greenland-Scotland Ridge and the marine system appears to be a ‘push-and-pull’ system rather than a seesaw system. ‘Pull’ during the warm interstadials, when convection in the Nordic seas was active; ‘push’ during the cold stadials, when convection stopped and warm water from the south-central Atlantic pushed northward gradually warming the North Atlantic and Nordic seas.

The climate of last glacial period was extremely unstable and interrupted by about 24 distinct warming and cooling events. The events are generally termed Greenland interstadials and stadials^1^ or Dansgaard-Oeschger events (D-O) and they are most prominent in the Greenland ice core records, where they consist of an abrupt warming to warm interstadial conditions followed by a more gradual cooling and a rapid drop to very cold stadial conditions^2^. The events are also recorded in the Antarctic ice cores, but the amplitudes here are smaller and the warmings are gradual in contrast to the abrupt warmings in the Greenland cores. The D-O events in the northern and southern ice cores are furthermore out of phase or even in anti-phase^3^,^4^,^5^.

Imprints of D-O events have widespread occurrences in the sediments and paleoceanographic records of the world oceans (Fig. 1). The strongest indications are from the North Atlantic and Nordic seas, where the imprints often resemble the pattern recorded in the Greenland ice cores with abrupt warmings and gradual coolings^6^,^7^ (Fig. 1). The primary cause for the climatic instability is accordingly attributed to changes in the rate of convection in the Nordic seas and North Atlantic, affecting the strength of the Atlantic Meridional Overturning Circulation (AMOC)^4^,^8^,^9^. At the beginning of the cold stadials, convection stopped or was severely reduced^10^,^11^,^12^,^13^. The result was a decrease in the northward transport of warm water and sudden cooling of the North Atlantic to very low temperatures and a warming of the South Atlantic^4^. Renewed convection at the beginning of the interstadials created the opposite effect. The out-of-phase relationship between the temperature fluctuations in the Greenland and Antarctic ice cores is often referred to as the bipolar seesaw or as a “southern lead” as the warmings seem to start earlier in the south than in the north^4^,^5^,^8^,^14^,^15^, although recent studies indicate that the actual temperature maxima occurred about 200 years earlier in Greenland than in Antarctica^16^.

Paleodata indicate that the changes in sea surface temperatures (SST) in the southern and central Atlantic followed the gradual warming pattern from the Antarctic ice cores^3^,^17^,^18^,^19^. Recent studies suggest this pattern continued all the way to the southern edge of the so-called IRD-belt^20^. This belt, which stretches across the Atlantic from Newfoundland to Ireland and Portugal, is characterized by glacial sediments containing distinct layers with abundant IRD reflecting periodical releases of huge numbers of icebergs from the Laurentide ice sheet^6^,^21^. These outbreaks, which are termed Heinrich events, are generally considered to be in phase with the larger and longer-lasting stadials in the Greenland ice cores^6^, although in the central northernmost Atlantic the arrival of icebergs may have lagged the beginning of the cold phase by several hundred years^13^.

While is generally accepted that the overall difference between the D-O oscillations in the northern and southern hemispheres is caused by variability in the AMOC, there is no consensus regarding the various processes that might have affected this variability and on how they interplayed with each other. Numerous factors have been suggested including changes in the strength^6^,^22^,^23^,^24^ and location of the deep convection^25^, changes in the continental ice sheets^26^, melt water release^9^, variability of sea ice cover^27^, heat exchange between the ocean and the atmosphere, and atmospheric heat transport^28^.

A major obstacle seems to be the scarcity of information from the North Atlantic between the IRD-belt and the Greenland-Scotland Ridge, where only a few studies have been carried out^13^,^29^,^30^,^31^,^32^. This area is important as it is close to the Nordic seas and Greenland ice cap and still represents the open Atlantic. Here we examine the configuration of D-O events 17–3 in core SO82-02GGC (SO2) taken at a water depth of 1730 m on the western side of the Reykjanes Ridge (Fig. 1). The core site is located north of the IRD-belt in an area showing relatively low influence of meltwater and ice. It is, therefore, ideally positioned for the examination of open ocean changes in the northernmost Atlantic^29^,^30^,^31^. We analyze the oscillation pattern of D-O events in both the surface and bottom water. We then compare the results with the corresponding events in the Greenland and Antarctic ice cores.

## Results

### Lithology

**Figure 1:**
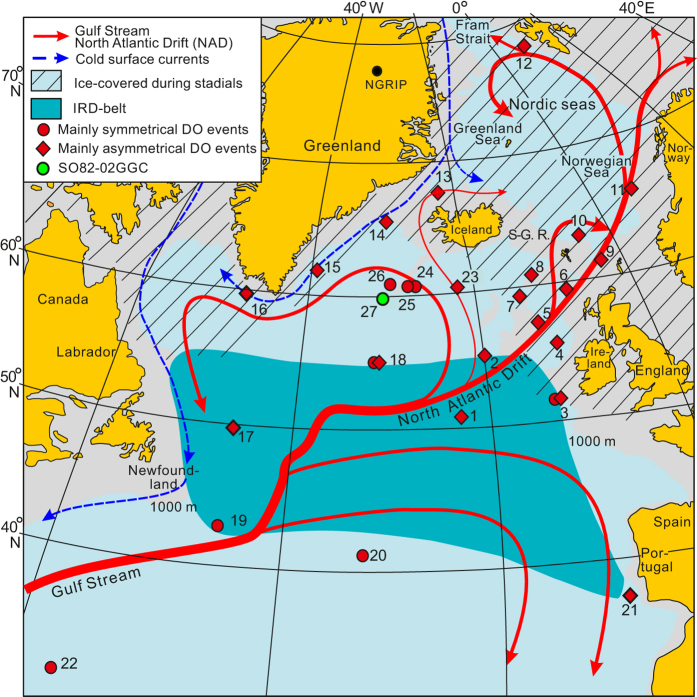
Map of the North Atlantic and Nordic seas showing location of core SO82-02GGC and examined published records. Modern major warm and cold surface currents are indicated. IRD-belt (ref. 21) is marked by darker blue color. Areas estimated to have been covered by sea ice during stadials outside of the IRD-belt are hatched. Records showing primarily abrupt warming at the stadial-interstadial transitions are marked by diamonds, while records showing primarily gradual warming are marked by circles. (The map was made using MapInfo Professional version 12 software, http://www.mapinfo.com/).

**Figure 2:**
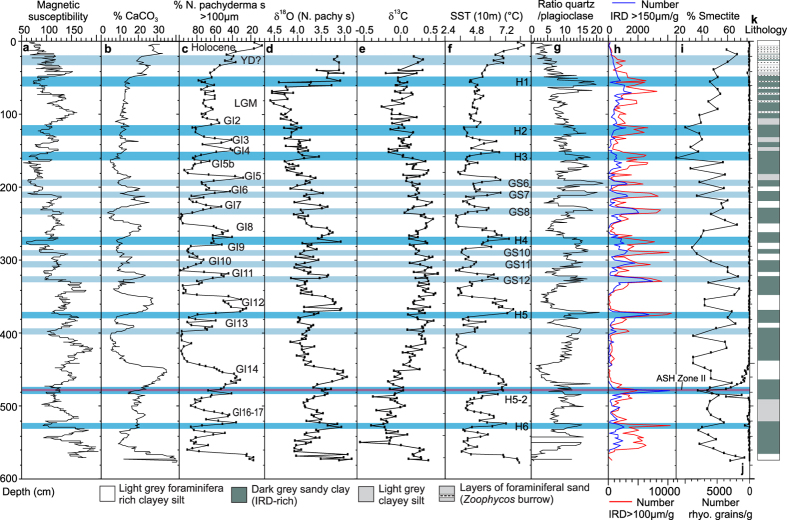
Stratigraphy, lithology and paleoceanographic proxies for core SO82-02GGC. (**a**) Magnetic susceptibility, (**b**) % CaCO3, (**c**) % *Neogloboquadrina pachyderma* s (>100 μm), (**d**) δ^18^O record measured in *N. pachyderma* s, (**e**) δ^13^C record measured in *N. pachyderma* s, (**f**) sea surface temperature SST at 10 m water depth calculated by transfer functions on the basis of planktonic foraminiferal faunas >100 μm, (**g**) quartz/plagioclase ratio, (**h**) concentration of IRD >100 μm and >150 μm, respectively, in number per gram dry weight sediment, (**i**)% smectite, (**j**) concentration of rhyolitic tephra per gram dry weight sediment, and (**k**) lithological log (see legend at bottom of figure). Light blue horizontal bars mark stadials, dark blue bars Heinrich events, and purple bar marks position of Ash Zone II. Greenland interstadial (GI) and stadial numbers (GS), and Heinrich events (**H**) are indicated. Data in columns (**a**,**b**,**g**,**i**) are from refs 30,32, the remaining data are from the present study.

The focus of the investigation is core SO2 taken during a cruise of RV Sonne in 1992 on the western side of the Reykjanes Ridge in the central North Atlantic (pos. 59°21.465N, 31°05.089W)^32^ (Fig. 1). The sediments consist of alternating layers of light grey, clayey silt and dark grey, sandy clay (Fig. 2k). The sedimentology of the core has previously been described, identifying D-O events 2–21^30^,^32^.

### Age model and correlation

The core top is dated to 7340 cal years BP indicating an early Holocene age. The upper 75 cm of the core is penetrated by zoophycos burrows and only dates from undisturbed samples from between the burrows are included in this study (Fig. 2k). Two dates performed on the planktonic foraminiferal species *N. pachyderma* s from 62.5 cm and 56 cm gave ages of 18.7 ka and 17.8 ka, respectively, which correlate in time with Heinrich event H1 (19–16 ka; e.g., ref. 6) (Table 1). In SO2, as elsewhere in the North Atlantic, H1 is characterized by a high concentration of IRD, high percentages of *N. pachyderma* s and low δ^18^O values (Fig. 2c,d,h).

Greenland stadials and interstadials (GI 17–GI 1) and Heinrich events H6–H1 have previously been identified in SO2 by Moros *et al.* (refs 30,32) on the basis of changes in the distribution of quartz, calcium carbonate and magnetic susceptibility (Fig. 2a,b,g). This identification is confirmed by the faunistic changes and the distribution of IRD described in this paper. Stadials in cores from the Reykjanes Ridge are normally distinguished by sharp peaks in the concentration of IRD^29^,^30^,^31^,^33^. In core SO2, these peaks coincide with low percentages of smectite in the clay fraction (Fig. 2g,h,i). The low smectite content is typical for the stadials in the Nordic seas and North Atlantic, where it reflects a reduction in the overflow from the Nordic seas^34^,^35^.

**Figure 3:**
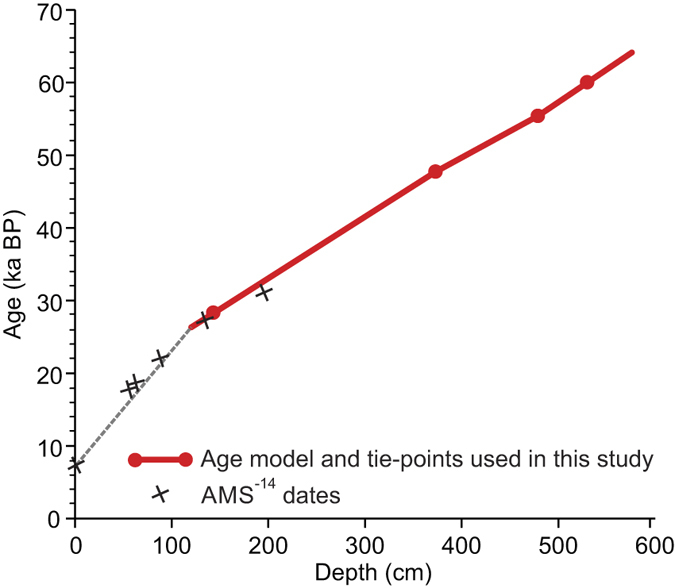
Age model for core SO82-02GGC. Age-depth plot for the interval investigated in the present study based on ages of four tie-points (red line and red dots). The model was extended to the core top using the calibrated AMS-^14^C age of the uppermost sample (grey dashed line). The positions of calibrated AMS-^14^C dates are indicated (see also Table 1). Due to uncertainty about the calibrated AMS-^14^C ages (partly because of bioturbation), the age model for the upper part of the core is shown only as a sketch.

We constructed an age model for the investigated interval of SO2 using the ages of four well-dated tie-points (Fig. 3). The youngest and the oldest of these tie-points are the transitions between Marine Isotope Stages (MIS) 3/2 and MIS 4/3 dated to 28,000 years and 60,000 years, respectively^36^. In SO2, high planktonic δ^18^O values and low δ^13^C values together with an AMS ^14^C date of 22.1 ka at 88 cm indicate that the interval 140–60 cm correlates with MIS 2. The MIS 3/2 transition is placed at 140 cm between Heinrich events H3 and H2 (Fig. 2) corresponding to the position indicated in nearby core ODP983 (Fig. 1) (ref. 13, Extended data Fig. 4). Similarly, high planktonic δ^18^O values combined with low δ^13^C values and the recognition of Heinrich event H6^30^,^32^ identifies MIS 4 and places the MIS 4/3 boundary at 530 cm (Fig. 2d,e). In between these two tie-points we use the NGRIP age of ASH Zone II dated to 55,400 years^37^. In SO2, ASH Zone II is recognized at 478 cm down-core as indicated by peaks in the number of rhyolitic and basaltic tephra grains (see ref. 30) (Fig. 2j). The last tie-point is placed at the rapid decrease in IRD marking the transition from Heinrich event H5 to interstadial 12 at 372 cm (Fig. 2h). This easily recognizable event is dated to 47,662 ± 1230 cal years BP (44,790 ^14^C years) in the extremely well dated core PS2644 from north of Iceland^38^. The age model was then calculated under the assumption of linear sedimentation rates between dating points with a small extrapolation at the lower end of the core (Fig. 3) (for a more detailed discussion of the age model see Methods section).

### Distribution of planktonic foraminifera and variability of water temperatures

**Figure 4:**
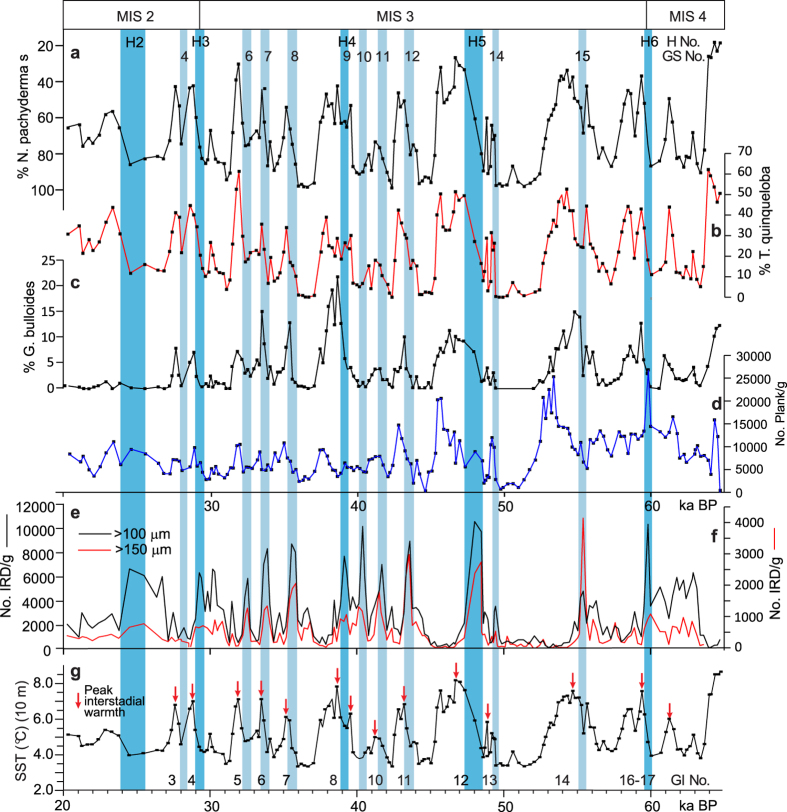
Selected paleoceanographic proxies for core SO82-02GGC shown on time scale 20–65 ka. (**a**) % *Neogloboquadrina pachyderma* s, (**b**) % *Turborotalita quinqueloba*, (**c**) % *Globigerina bulloides*, (**d**) Total number of planktonic foraminifera per gram dry weight sediment, (**e, f**) concentration of IRD >100 μm and >150 μm, respectively, in number per gram dry weight sediment, (**g**) sea surface temperature SST at 10 m water depth calculated by transfer functions on the basis of planktonic foraminiferal faunas >100 μm. Red arrows indicate position of maximum interstadial temperatures. Top bar shows marine oxygen isotopes stages (MIS 4–1). All data are plotted versus calendar age. Greenland interstadial (GI) and stadial numbers (GS), and Heinrich events (H) are indicated.

Faunistically, the peak interstadials are distinguished by low percentages of the polar planktonic foraminiferal species *N. pachyderma* s and high percentages of species characterized as subpolar species, in particular *Globigerina bulloides* and *Turborotalita quinqueloba*^39^ (Fig. 4a,b,c). Similar faunas predominate the interstadials in nearby core SO82-05GGC^29^ and in other cores from the North Atlantic and Nordic seas.

**Figure 5:**
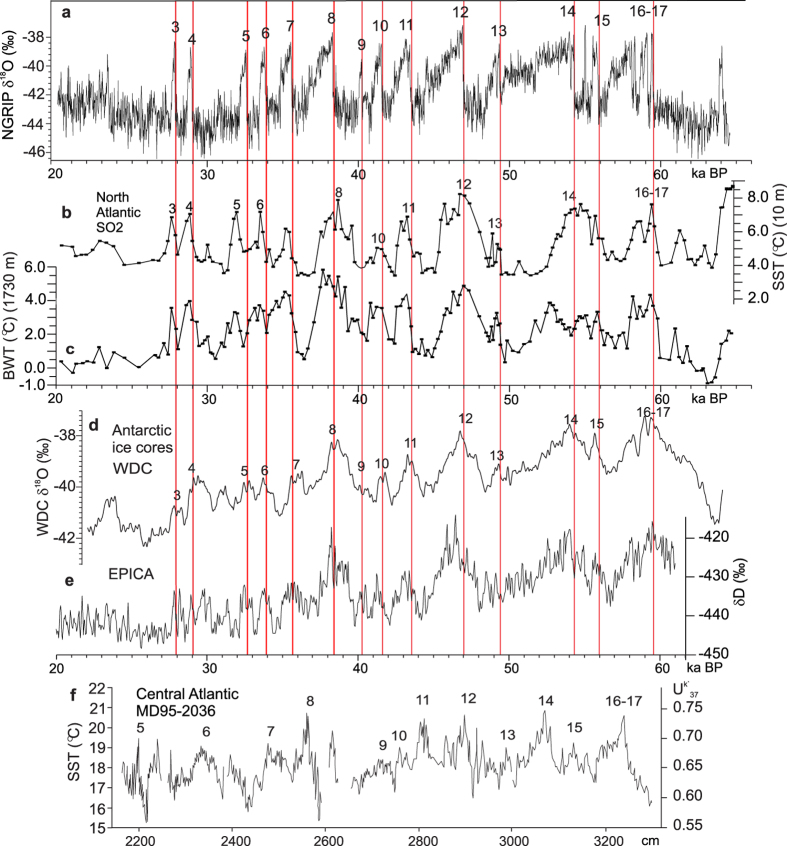
Glacial SST variability for core SO82-02GGC compared to Arctic and Antarctic climate proxies and SST for marine core MD95-2036. (**a**) δ^18^O record from Greenland NGRIP ice core on GICC05 time scale^37^. (**b**) Sea surface temperatures at 10 m water depth for marine core SO82-02GGC plotted versus calendar age. (**c**) Bottom water temperatures at 1730 m water depth for marine core SO82-02GGC plotted versus calendar age. (**d**) δ^18^O record for Antarctic ice core WDC calculated to AICC12 time scale (~=NGRIP GICC05 time scale)^16^. (**e**) δD record for Antarctic ice core EPICA plotted versus GICC05 time scale^14^. (**f**) Alkenone sea surface temperature from marine core MD95-2036^18^ plotted versus core depth (cm). For location of core MD95-2036 see Fig. 1, no. 22). Red lines indicate position of stadial/interstadial boundaries.

In order to estimate surface and bottom water temperatures we have analysed the distribution of planktonic and benthic foraminifera using transfer functions (see Methods). The interstadial SST vary typically from 7–8.5 °C as compared to 3–3.5 °C for the stadials (Figs 4g and 5b,c). Bottom water temperatures are generally between 3 °C and 3.5 °C lower than the SST (Fig. 5c). The transition from cold stadial to warm interstadial conditions and vice versa is mostly gradual. The average duration of the warming periods is about 800 years (see Methods and Table 2).

## Discussion

### Interstadial and stadial conditions over Reykjanes Ridge

In nearly all D-O events, maximum temperature occurs in the early part of the interstadials (Fig. 4g). The peaks are marked by low percentages of the polar planktonic foraminiferal species *Neogloboquadrina pachyderma* s, high percentages of subpolar species and high abundance of planktonic foraminifera (Fig. 4a–d). The interstadial SST between ~6.5 °C and ~8.5° are close to interstadial temperatures estimated from transfer functions and Mg/Ca ratios for nearby cores SO82-05GGC^29^, LO09-18 and DS97-2P^31^ and only slightly lower than the present temperatures in the area (Figs 2f and 4g). The low concentration of IRD indicates an almost total lack of sea ice and icebergs in the area (Fig. 4e,f).

Halfway through most interstadials, the planktonic faunas change rapidly reflecting a significant temperature drop, and the later part of the interstadials (interstadial cooling phase^2^) were generally very cold with low abundances of planktonic foraminifera (Fig. 4a–d,g). Yet, the concentration of IRD remains low indicating that the number of icebergs did not increase. The cooling is in accordance with the δ^18^O record, which shows increasing values coinciding with the decreasing temperatures (Fig. 2d). Jonkers *et al.* (ref. 31) noticed a similar rapid cooling during interstadial GI 8 in nearby cores LO09-18 and DS97-2P. In fact, a prolonged period with cold surface water and slow convection before the arrival of IRD has been documented in several cores from the North Atlantic north of the IRD-belt^10^,^13^,^29^.

The abrupt increase in IRD at the beginning of the stadials indicates a rapid growth in sea ice cover and in the number of melting icebergs^29^,^30^,^31^,^33^ (Figs 2h and 4e,f). According to ref. 29, most of the IRD on the Reykjanes Ridge are from Eastern Greenland and delivered by the East Greenland Current. Sea surface temperatures, as estimated from transfer functions, remained very low and often reached a minimum at the interstadial-stadial transitions. From this point we see a decrease in *N. pachyderma* s and an increase in *G. bulloides* and *T. quinqueloba* indicating a warming of the surface water, and the transfer functions calculate an increase in summer SST from 3.4 °C at the beginning of the stadials to about 7.0 °C at the transition to the next interstadial. Several studies have shown that the sudden decline in IRD at the end of the stadials signifies the disappearance of sea ice and icebergs from the Nordic seas and northernmost Atlantic and coincides with the resumption of convection in the Nordic seas and the abrupt temperature increase in the Greenland ice cores^2^,^22^,^23^,^24^,^40^.

However, in SO2, sea surface temperatures continued to increase and peak warmth were, on average, first reached about 200 years into the interstadials (Fig. 4g; Table 2). The average duration of the total interstadial warming period is close to 800 years as compared to 40 years for the atmospheric shifts in the NGRIP ice core (Table 2). Gradual surface and subsurface warming during stadials/Heinrich events over the Reykjanes Ridge has previously been demonstrated for H4 based on Mg/Ca^31^. The results from SO2 indicate that gradual surface warming occurred during all stadials/Heinrich events between c. 65 and 25 ka (Figs 4g and 5b,c). The relatively low δ^13^C values suggest poorer subsurface ventilation as compared to the interstadials (Fig. 2e).

The fluctuations in bottom water temperatures are an almost exact repetition of the fluctuations in the surface water, showing the same pattern of gradual warmings and coolings (Fig. 5b,c; Table 2). The similarity indicates a close coupling over the Reykjanes Ridge between surface water and intermediate water and a homogenous water column down to a depth of at least 1730 m.

### The stadial-interstadial transitions in the Atlantic Ocean

Comparing the development of the D-O events over the Reykjanes Ridge with other records from the North Atlantic and Nordic seas it appears that the warming of the intermediate water was gradual throughout the North Atlantic and Nordic seas during all stadials and Heinrich events. Gradual warming of the intermediate water during stadials/Heinrich events and a slowdown of the AMOC was first proposed in 1996 for a core from the southern Nordic seas^40^ and it has later been corroborated in numerous studies from the North Atlantic and also in model experiments^22^,^23^,^41^,^42^,^43^,^44^,^45^. In contrast to the ubiquitously occurring gradual warming of the intermediate water, the warming of the surface and subsurface water shows significant local differences. A survey of previous studies from the North Atlantic realm indicates that the large central part of the northern Atlantic between the IRD-belt and the Greenland-Scotland Ridge experienced gradual warming similar to the warming pattern over the Reykjanes Ridge, while abrupt warming was limited to the Nordic seas, the IRD-belt and land-near areas farthest to the northeast and northwest (Fig. 1; Table 3).

Paleoceanographically, it appears that gradual surface warming occurred in open marine areas with low influx of meltwater and modest amounts of sea ice and icebergs, while abrupt warming occurred in areas with a large influx of meltwater, numerous icebergs and an extensive ice cover. The conditions promoting abrupt warmings have been examined in detail in several studies from the Nordic seas. During stadials, large numbers of melting icebergs^40^ and sea ice created a stratified water column composed of a relatively thin layer of cold, low saline surface water overlying a denser intermediate water mass, which was gradually warming^22^,^40^. Similar conditions probably existed in the IRD belt during Heinrich events^6^,^21^. In the Nordic seas, the abrupt warming has been attributed to a rapid surfacing of the warm intermediate water, which broke the stratification and restored convection^22^.

The results from SO2 add some significant details to this scenario. In SO2, the gradual warming begins simultaneously with or slightly before the abrupt rise in ice rafting (Fig. 4f,g). This indicates to us that the increased melting of icebergs was caused by the warming. Warming in connection with stadials/Heinrich events has previously been suggested to cause ice melting and increased discharges of icebergs^43^,^46^. The melting lead to a higher input of meltwater, and in the Nordic seas and IRD-belt, where icebergs were more numerous, the upper ocean became stratified.

The average temperature for the bottom water in SO2 (Fig. 5c) follows roughly previous estimates for intermediate-water temperatures in the North Atlantic and southern Nordic seas^22^,^41^,^42^,^43^. This suggests that the temperature fluctuations in SO2 reflect the general temperatures of the Gulf Stream system^18^, which again is controlled by the temperatures of the central and southern Atlantic (Fig. 5f). This implies that the similarity between the D-O events in SO2 and the events in the southern Atlantic and in the Antarctic ice cores is the result of a direct southern influence on the paleoceanography of the northern Atlantic during D-O events^23^,^24^,^33^,^47^.

Furthermore, new evidence from Antarctic ice core WDC indicates that the interstadial warmings in SO2 and in the Antarctic ice core probably were synchronous (Fig. 5b,d,e). Precise correlation between core WDC and the Greenland NGRIP ice core indicate that the maximum interstadial temperature in WDC on average occurred 218 years after the abrupt warming over Greenland^16^. Our calculations indicate that the maximum temperature in SO2 on average occurred 211 years after the start the interstadial (Table 2). The similarity of these figures strongly indicates that maximum interstadial warmth was reached practically simultaneously in core WDC and core SO2. The synchronicity of maximum interstadial warmth combined with the overall similarity of the D-O events in WDC and SO2 (Fig. 5b,d) indicate further that the gradual warmings at the stadial-interstadial transitions occurred synchronously throughout the Atlantic Ocean. Only the subsurface penetration into the Nordic seas was possibly slightly delayed.

### Implications

The results of this study indicate that the D-O warmings in the open North Atlantic were gradual and in phase with the gradual warmings in the Antarctic ice cores and in the southern and central Atlantic. They also indicate that the warmings were out of phase with the abrupt warmings in the Greenland ice cores, in the Nordic seas, and areas in the North Atlantic strongly affected by meltwater during stadials. This implies that the hinge line between areas showing gradual warming and areas showing abrupt warming was displaced far to the north close to the Greenland-Scotland Ridge. Considering this geographical asymmetry, the term “bipolar seesaw” seems confusing with respect to marine conditions. This is underlined by the fact that the main cause for D-O events appears to be the ‘turn-on’ and ‘turn-off’ or a slow-down of convection in the Nordic seas^4^,^10^,^11^,^12^,^13^,^25^ with the southern Atlantic reacting mainly passively^4^. It would be more accurate to describe the system as a ‘push and pull’ system. ‘Pull’ during interstadials, when convection in the Nordic seas was active and the AMOC strong and ‘push’ during stadials, when convection stopped or slowed.

The driving forces for the interstadial Atlantic circulation system were undoubtedly the same as at present, when 75% of the inflow to the Nordic seas is returned to the Atlantic as cold deep water overflowing the Greenland-Scotland Ridge^48^. In the Atlantic, the overflow water enters the NADW and becomes a very important component of the AMOC^49^. The overflow creates a sea level gradient (barotropic pressure gradient) across the Greenland-Scotland Ridge pulling warm Atlantic water into the Nordic seas^48^. The gradient is an important part of the forcing for the inflow to the Nordic seas, and a reduced overflow can be expected to create a corresponding reduction in the inflow^48^.

The stadial circulation system has no modern analogue, but it is generally agreed that the AMOC was weak and warm water from the central and southern Atlantic ‘pushed’ northwards gradually warming the North Atlantic^3^,^4^. This study shows that in the northernmost North Atlantic the warming coincides with sharp increase in deposition of IRD implying increased ice rafting, increased melting of icebergs, and increased spreading of meltwater. In the Nordic seas and IRD-belt the result was a stratified ocean and the development of very cold stadial conditions.

## Methods

### Fauna analysis and ice rafted material

The core was sampled in 0.7 cm thick slices at approximately 1, 2, or 3 cm intervals according to changes in lithology and colour. The samples were weighed, dried and weighed again and subsequently sieved over 63 μm and 100 μm sieves. The residues were dried and weighed. More than 300 specimens of planktonic and benthic foraminifera were picked, counted and identified from the >100 μm size fractions. Mineral grains (excluding volcanic material), supposedly representing ice rafted debris (IRD), were counted in the >100 μm size fraction and concentrations calculated. Basaltic and rhyolitic grains were counted and calculated separately. The samples were subsequently dry sieved over mesh size 150 μm. Mineral grains including blocky and heavy basaltic material (ice rafted), but excluding porous basaltic and rhyolitic shards (potentially airborne or current distributed) were picked and counted. The concentration of IRD >150 μm was calculated.

### Temperature calculations

Sea surface (SST) and bottom water temperatures (BWT) were estimated by transfer functions using the C2 program^50^. The SST calculations were based on the 100 μm size fraction of planktonic foraminifera. We applied the WAPLS (Weighed Average Partial Least-Squares) method using one component following the recommendations of Birks (ref. 51). The calculations were based on the database of Hald and Husum (ref. 52) of the distribution of planktonic foraminifera in the >100 μm size fraction. We extended this database to include samples from both colder and warmer areas using published data^53^,^54^. The modern ocean temperatures were taken from the *World Ocean Atlas (1998)*^55^. Temperatures were calculated for a water depth of 10 m. For the BWT calculations we used a modified and extended version of the database of the distribution of living benthic foraminiferal faunas in the Nordic seas published by Sejrup *et al.* (ref. 56), omitting samples from water depths of less than 250 m and adding new data from the North Atlantic Ocean^43^. The additional material consists of previously published records on the distribution of live benthic foraminifera in the southern Nordic seas and in the North Atlantic Ocean comprising the depth interval 250–~2000 m (see also ref. 43). The supplementary data are from off Ireland, Bay of Biscay, off Portugal, the central North Atlantic Ocean, and the east coast of Canada and the United States (see references in ref. 43). We applied the WAPLS (Weighed Average Partial Least-Squares) method using one component following the recommendations of Sejrup *et al.* (ref. 56). The calculations were based on ocean temperatures from the *World Ocean Atlas (2009)*^57^ or temperatures reported in the foraminiferal investigations.

The duration of the interstadial warming periods in the NGRIP ice core (Table 2) was estimated using the NGRIP δ^18^O data^37^. The duration of the warming periods in SO2 was estimated using the planktonic and benthic water temperatures calculated by transfer functions and the age model presented in Fig. 3.

### Isotope analysis

Oxygen isotope analyses were performed on the planktonic species *Neogloboquadrina pachyderma* s at the GMS laboratory of the Bjerknes Centre for Climate Research at the University of Bergen on a Finnigan MAT 251 mass spectrometer equipped with an automatic “Kiel device” preparation line (in 2003). Foraminiferal tests of size fraction 150–250 μm (typically 6–10 specimens) were crushed and cleaned in an ultrasonic bath before analyses. The reproducibility of oxygen isotope measurements is ±0.07‰ based on replicate measurements of carbonate standards. All δ^18^O results are reported in ‰ vs. PDB, using NBS 19 as the standard.

### Radiocarbon dating

Radiocarbon dates by accelerator mass spectrometry (AMS-^14^C dates) were carried out on monospecific samples of the planktonic foraminifera *N. pachyderma* s or *Globigerina bulloides* (Table 1) at the Leibniz-Laboratory for Radiometric Dating in Kiel (KIA labels) and at Aarhus University (AAR labels). The AMS-^14^C dates were calibrated using the Calib7.02 program and the Marine13 conversion using reservoir ages of 405 years inherent in the program^58^ (Fig. 3).

### Age model

The age model used in the present study is based on four well-dated tie-points (Fig. 3). The age-depth plot indicates a uniform sedimentation rate throughout the investigated time period. In order to examine this interpretation we have compared our age model with a second age model created by tuning the SO2 record to the NGRIP GICC05 time scale^37^. The tuning was obtained by tying the abrupt shifts in the concentration of IRD at the beginning and end of the stadials in SO2 to the stadials in the ice core providing a total of 28 tie-points. Fine-tuning of details in D-O events in marine and Greenland ice core records often changes the original configuration of the events in the marine records towards the pattern seen in the ice core. For example, a gradual warming pattern may shift to an abrupt. The changes are then attributed to variations in the sedimentation rate in the marine record.

**Figure 6:**
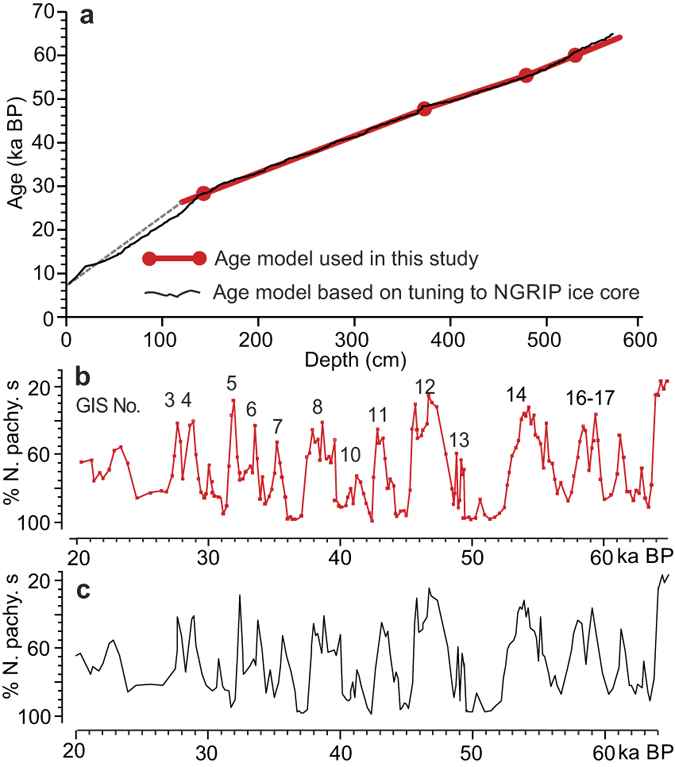
Tuned and un-tuned age models for core SO82-02GGC and abundance plots of *Neogloboquadrina pachydermas*. (**a**) Red line and red dots show un-tuned age model as used in the present study (see Fig. 3). Black line show age model based on tuning of abrupt rise and fall in IRD concentration to NGRIP ice core ages (GICC05 time scale; ref. 37). (**b**) Percent abundance of *N. pachyderma* s plotted versus un-tuned age model. (**c**) Percent abundance of *N. pachyderma* s plotted versus tuned age model.

However, such changes do not occur in the records of SO2 as indicated by plots of the percent abundance of *N. pachyderma* s, which are very similar irrespective of whether they are plotted on the un-tuned or the tuned age scales or, in fact, on a cm scale (Figs 2c and 6b,c). The age-depth plots demonstrate also that within the investigated interval the two age models are almost identical (Fig. 6a). We interpret the similarity of the two age models and the close match between the un-tuned, tuned and cm-scaled plots as an indication of a very uniform sedimentation rate in SO2 with negligible differences between stadials and interstadials. Only in the uppermost part of the core above c. 120 cm we notice a significant change in sedimentation rate (Fig. 6a).

### Gradual warmings versus abrupt warmings

The cores shown in Fig. 1 and Table 3 contain sea surface temperature reconstructions from the North Atlantic and Nordic seas during D-O/Heinrich events. We have limited our examination to reconstructions based on percentage of *N. pachyderma* s, transfer functions applied to planktonic foraminiferal faunas, Mg/Ca ratios in planktonic foraminifera, alkenones, and δ^18^O values measured in planktonic foraminifera (Table 3). We have examined the Dansgaard-Oeschger events in each core and subdivided the cores into two groups based on the warming-cooling pattern of the events: (1) Cores showing an asymmetrical pattern similar to the saw-tooth pattern in the Greenland ice cores and (2) cores showing a symmetrical pattern similar to the pattern seen in the Antarctic ice cores. Some records show both symmetrical and asymmetrical patterns. These records are classified according to the pattern shown by the majority of events. Cores with insufficient resolution to distinguish a clear pattern were omitted. A few records were interpreted as less dependable because of a very close tuning to the Greenland ice cores and also omitted. However, it should be noted that the tuning problems can sometimes be obviated if the data also are available on the original depth scale. For areas with a good coverage of cores we only show a single or a few cores evaluated as representative.

## Additional Information

**How to cite this article**: Rasmussen, T. L. *et al.* North Atlantic warming during Dansgaard-Oeschger events synchronous with Antarctic warming and out-of-phase with Greenland climate. *Sci. Rep.* **6**, 20535; doi: 10.1038/srep20535 (2016).

## Figures and Tables

**Table 1:** AMS ^14^C dates from core SO82-02GGC.

Depth cm	^14^C age^1^	Error (1σ)	Lab. Code	Species	Cal ages	Error (1σ)
1	6820	130	AAR1389	*G. bulloides*	7340	120
56	15,059	80	KIA18594	*N. pachy*. s	17,824	113
62.5	15,780	45	KIA18595	*N. pachy*. s	18,654	61
88	18,660	110	KIA18597	*N. pachy*. s	22,124	155
134	23,340	270	AAR1391	*N. pachy*. s	27,265	265
192	27,060	430	AAR1392	*N. pachy*. s	30,814	341

^1^Conventional ages.

**Table 2:** Estimated duration in years of Dansgaard-Oeschger warmings in marine core SO82-02GGC at surface (10 m) and bottom (1730 m) compared to NGRIP ice core.

Interstadial	SO82-02GGC			NGRIP
	10 m	1730 m	Interstadial warming^1^	Duration
GI3	415	415	325	78
GI4	795	1470	310	58
GI5	485	640	275	39
GI6	830	390	-80	48
GI7	795	1270	75	41
GI8	690	1270	120	32
GI9	725	1375	–	24
GI10	930	900	110	29
GI11	860	760	20	20
GI12	1625	1540	630	39
GI13	690	590	170	32
GI14	810	–	380	35
GI17	770	770	200	34
Average	802 ± 79	949 ± 120	211 ± 54	39 ± 4

^1^Duration of surface warming in years from drop in IRD (=stadial/interstadial boundary) to interstadial peak warmth (see text for explanation and Fig. 4g).

**Table 3:** List of cores indicated in Fig. 1 showing core names, temperature proxy used in the evaluation of D-O configurations, authors and reference numbers.

Core no. in Fig. 1	Core	Proxy for temperature	References
1	DSDP609	% *N. pachyderma* s	Bond *et al.* (ref. 6)
2	VM23-81	% *N. pachyderma* s	Bond *et al.* (ref. 6)
3	MD01-2461	Mg/Ca *N. pachyderma* s and *G. bulloides*	Peck *et al.* (ref. 59)
4	MD04-2822	% *N. pachyderma* s	Hibbert *et al.* (ref. 60)
5	NA87-22	SST, transfer functions planktic foraminifera	Elliot *et al.* (ref. 61)
6	MD04-2829CQ	% *N. pachyderma* s	Hall *et al.* (ref. 62)
7	DAPC-02	% *N. pachyderma* s	Rasmussen and Thomsen (ref. 53)
8	ENAM33	% *N. pachyderma* s	Rasmussen and Thomsen (ref. 22)
9	LINK17	% *N. pachyderma* s	Rasmussen and Thomsen (ref. 53)
10	ENAM93-21	% *N. pachyderma* s	Rasmussen and Thomsen (ref. 22)
11	ODP644	δ^18^O *N. pachyderma* s	Fronval *et al.* (ref. 63)
12	JM05-031GC	% *N. pachyderma* s	Rasmussen *et al.* (ref. 42)
13	PS2644	δ^18^O *N. pachyderma* s	Voelker *et al.* (ref. 38)
14	JM96-1225	% *N. pachyderma* s and *G. bulloides*	Hagen and Hald (ref. 64)
15	SU90-24	δ^18^O *N. pachyderma* s	Elliot *et al.* (ref. 61)
16	P-012, P-013	δ^18^O *N. pachyderma* s	Stoner *et al.* (ref. 65)
17	P-094, MD95-2024	δ^18^O *N. pachyderma* and *G. bulloides*	Hillaire-Marcel and Bilodeau (ref. 66)
18	JPC-13	% *N. pachyderma* s, δ^18^O *N. pachyderma* s	Hodell *et al.* (ref. 33)
19	CH69-K09	SST, transfer functions planktic foraminifera	Labeyrie *et al.* (ref. 67)
20	U1313	Alkenone temperature	Naafs *et al.* (ref. 20)
21	MD95-2042	δ^18^O *G. bulloides*	Shackleton *et al.* (ref. 23)
22	MD95-2036	Alkenone temperature	Sachs and Lehman (ref. 18)
23	V29-202	% *N. pachyderma* s	Oppo and Lehman (ref. 68)
24	ODP983	% *N. pachyderma* s	Barker *et al.* (ref. 13)
25	DS97-2P, LO09-18	Mg/Ca *N. pachyderma*s, % *N. pachyderma* s	Jonkers *et al.* (ref. 31)
26	SO82-05GGC	SST transfer functions planktic foraminifera, % *N. pachyderma* s	van Kreveld *et al.* (ref. 29)
27	SO82-02GGC	SST transfer functions planktic foraminifera, % *N. pachyderma* s	This study
